# The Value of Hydrocyanate of Iron in the Treatment of Neuralgia

**Published:** 1881-12-20

**Authors:** J. Thos. Stovall

**Affiliations:** Columbia, Ala.


### THE VALUE OF THE HYDROCYANATE OF IRON IN THE TREATMENT OF NEURALGIA.

BY J. THOS. STOVALL, M. D., OF COLUMBIA, ALA.

I desire to call the attention of the profession to the value of the hydrocyanate of iron in the treatment of neurilgia.

At this advanced period of medical science, there is constantly some new remedy being extolled by some of the medical profession as a specific or very nearly so, for some disease or class of diseases. Each new remedy has been tested and so often been found perfectly inert, that we are slow to grasp at every new preparation that is being lauded to the skies, as so curative and yet so palatable, etc. Yet we should not ride rough shodden over the bounds of reason and common sense, for a progressive science like medicine cannot discard everything new and cling to the old.

I wish to make a few suggestions upon the therapeutics of a remedy that I have tried frequently and thoroughly.

If I were to speak upon hypothesis I would say a great deal more than I intend saying in this article. A single fact in therapeutics is worth a thousand hypotheses.

The first I ever knew of the hydrocyanate of iron was in the latter part of 1879. It was shown and recommended to me by my friend, Dr. H. H. Christian, of this place, who has been using it in the treatment of neuralgia for the past seven or eight years, and with uniform success. Having noticed its effects in some obstinate cases of neuralgia, I determined to give it a thorough trial. Another reason why I felt such an interest in it was because I had never heard of the medicine before, as it is not officinal. I gave it a thorough trial, and could now hardly do without it in the treatment of neuralgia, especially in obstinate cases.

All physicians who have ever used it, have used it at or through the suggestion of Dr. C., as none of them had, like myself, ever heard of the drug. He first noticed its value set forth in Tilden & Co's *Materia Medica Journal* of some years ago, and he gave it a trial, and being so highly pleased with it, he thoroughly tested it, and still continues to use it.

I have been using it myself since 1879, and will never consider my *armamentarium* complete without it.

I have never used it in a single case but that I either effected a cure or gave more relief than by any other remedial agent. I will say that any and all forms of neuralgia are benefitted by it, whether due to a local or constitutional cause.

It is an anodyne, a hypnotic and an analeptic tonic. It also possesses highly sedative properties.

1st. It is most beneficial in cases of neuralgia dependent upon, or connected with an anæmic condition of the system.

In such cases the iron builds up the system by enriching the red globules of the blood, thus removing the prime factor in its production, the hydrocyanic acid relieving the morbid sensibility of the nervous system, giving us, in my estimation, a remedy *par excellence* in such cases.

2d. It is next most beneficial in those obstinate cases of neuralgia assuming periodicity. I consider the hydrocyanate an anti-periodic equal to or superior to the ferrocyanate.

Allow me to digress a little here. I know malaria has been assigned as the cause or a factor in the production of nearly, if not all, the diseases flesh is heir to, even the malarial belly-aches, yet I am thoroughly convinced that malaria is a great cause of neuralgia. Periodic neuralgia can be assigned to no other cause.

"Well," some would say, "quinia is my treatment." Of course they would say so, for quinia is given in nearly every disease. Why, I do not know. More as a force of habit (?) than anything else, I suppose. Why give quinia when we have a drug equally as potent at far lsss the cost? It is because it is the fashion, the weakness of the profession. In such cases quinia is a useful adjunct. The formula I generally use is as follows—

℞ Quinia, .................................. grs. xij,
Hydrocyanate of iron, .................... grs. xviij,
Morphia, .................................. gr. j.

Ext. gentian vel valerian q. s., make pillular mass, make 12 pills.
Sig. Take 1 pill every three hours till 6 are taken, wait 6 hours and take the balance.

I use this formula also in cases of acute idiopathic neuralgia.

In chronic cases I combine with the hydrocyanate, strychnia and belladonna (extr. of belladonna.)

I will cite some cases in which I have used it:

Case I. Lucy C——, æt. 13 years 4 months, was taken sick April 1st, with a light form of intermittent fever, with very acute pain over left eye, radiating through forehead and face. Very anæmic. Suffers greatly from nervousness and muscular twitchings. Experiences great inconvenience from nausea and vomiting. It is so great that it is with difficulty that she retains anything at all upon her stomach, (even nourishment). Appetite bad, bowels loose. Suffers from insomnia.

I applied a counter-irritant over epigastrium. Administered tartrate of iron and potassa, and quinia and morphia.

Under this treatment she experienced partial relief, still suffering at times with neuralgic pains.

She was taken with the measles the second week in May; all the above symptoms returning in aggravated form.

Recovered from measles, but relapsed June 24th. Appetite bad, bowels loose, stools dark, no fever; but suffered with excruciating pain over left eye, coming on by paroxysms, at times being almost unbearable. Only sleeps when under the influence of a hypnotic; very anæmic, skin of a marble color; could retain nothing upon the stomach; tongue pale, flaccid and dentated; pupils dilated: pulse quick, weak and frequent.

To relieve nausea and vomiting, applied counter-irritation to the stomach. I used several of our staunch remedies—belladonna, hyoscyamus, quinia, morphia, valerianate of bismuth, etc. All proved valueless only for the time being. The trouble yielded to hydrocyanate. Entirely well and has not been troubled since the 30th of June.

Case II. Mrs. W——, æt., 50 years, was taken with measles (broken out) July 7th, 1881. She suffered intensely from bronchitis. Bowels not troublesome. Suffered also with severe neuralgia; was called July 14th. Neuralgia pain began in left eye and extended to face. Suffered also with terrible reflex cough and pain in side. I put her, 15th of July, on the hydrocyanate, and on the 18th she was almost entirely well.

Case III. Mrs. H——. Had suffered with neuralgia for 2 or 3 years. Under the hydrocyanate, ext. nux. vom. and ext. belladonna, she entirely recovered. The formula was as follows—

℞ Hydrocyanate of iron,........................ ʒi,
Ext. nux vomica al,......................... grs. xv,
Ext. belladonna,............................ grs. viij.
Ext. gentian, q. s. ad pillular mass.
Make 60 pills. Sig. Take 1 pill before each meal.

I could cite numerous other cases but it would be useless.

From the administration of the hydrocyanate, the patient suffers no inconvenience. We have no depression or unpleasant feelings from its use, as from most drugs used in such cases. My assertions upon the use of this drug are based upon a thorough trial, and not a series of hypotheses. I do not pretend to say that it is a specific in neuralgia, but it comes nearer such than any remedy in our vast Materia Medica.

In conclusion I would say I would like the medical profession to give it a thorough trial, and report in this journal. I have never seen any article upon this drug in any journal, therefore would like to know the opinions of others. No text-book mentions it as I have seen. I desired to give the history of this drug, but could obtain nothing as to who first used it, or how long it has been used. or its merits are not appreciated; else we should see more of it in the current medical literature of the day. Dr. C. has used it for the past seven or eight years with uniform success. This article is not the one I had intended first for publication, but is merely a summary. Hope to hear from it early.—*American Medical Bi-Weekly.*

